# A case of an incidentally discovered epithelioid haemangioendothelioma

**DOI:** 10.1093/jscr/rjaf917

**Published:** 2025-11-23

**Authors:** Sabrina B J Belfi, Beata Bode-Lesniewska, Ilhan Inci

**Affiliations:** University of Nicosia Medical School, Makedonitissas Avenue, Nicosia CY‐2417, Cyprus; University of Zurich, Rämistrasse, Zurich 8006, Switzerland; Lucerne Cantonal Hospital, Institute of Pathology, Spitalstrasse, Lucerne 6000, Switzerland; University of Nicosia Medical School, Makedonitissas Avenue, Nicosia CY‐2417, Cyprus; University of Zurich, Rämistrasse, Zurich 8006, Switzerland; Klinik Hirslanden Zurich, Centre for Surgery Zurich, Thoracic Surgery, Witellikerstrasse, Zurich 8032, Switzerland

**Keywords:** thoracic surgery, epithelioid haemangioendothelioma, malignant pleural effusion

## Abstract

We report a case of a 73-year-old male with non-Hodgkin’s lymphoma who underwent a restaging CT during treatment, which raised concern for a coexisting pathology. Consequently, a CT-guided biopsy was performed and a diagnosis of a second malignancy, an epithelioid haemangioendothelioma (EHE), was made. This is an exceptionally rare finding, given that the estimated incidence of EHE is only 0.4 cases per million per year. The patient has multifocal disease, with multiple pulmonary nodules and paravertebral/intercostal masses. Treatment with pazopanib was initiated early on due to the patient’s significant systemic symptoms and recurrent malignant pleural effusions (MPEs). Definitive pleural interventions, including talc pleurodesis and indwelling pleural catheter placement, were provided but were ultimately unsuccessful, and the effusion became loculated. The patient continues to suffer from extreme breathlessness. Given the significant impact on the quality of life of patients, this report delves into the complex management of MPEs in EHE.

## Introduction

Epithelioid haemangioendothelioma (EHE) is an ultra-rare sarcoma exhibiting endothelial differentiation [1] with an incidence of only 0.4/10^6^/year [2]. Typically, EHE behaves as a low-grade malignancy; nonetheless, it has a strong tendency for systemic involvement [1], which is associated with a lower overall survival [3]. EHE may involve various regions of the body; the most frequent sites include the liver and the lungs [4]. By detailing the clinical course, this manuscript aims to contribute to and enrich the limited literature on EHE.

## Case report

A 73-year-old male with non-Hodgkin’s lymphoma (NHL) underwent a restaging CT, following three cycles of a rituximab-bendamustin regimen. Despite clear regression of the histologically NHL-confirmed retroperitoneal and mesenteric masses, the CT revealed that several lesions seen on initial CT scans, including the right paravertebral mass at T11/12, had remained constant in size. A new minor pleural effusion was also noted, raising concern for a coexisting pathology. The patient reported dyspnoea and pleuritic chest pain but denied any cough or haemoptysis. Constitutional symptoms included generalized right-sided pain, possibly originating from T11/T12 nerve root compression, significant weight loss (~30 kg over the past 1.5 years), irregular night sweats, and extreme fatigue. His past medical history includes hypertension. He has no family history of vascular tumours or cancers of the liver, lung, bone, soft tissue, or pleura. The patient does not currently smoke; however, he previously smoked, accumulating 7.5 pack-years.

**Figure 1:**
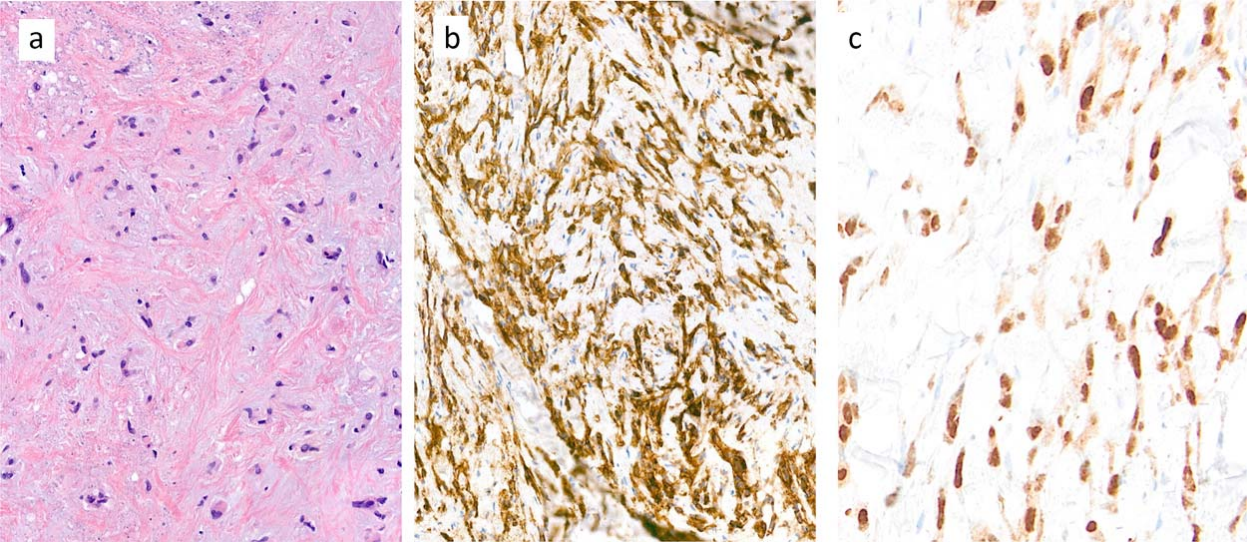
Core biopsy demonstrated a collagen-rich tumour with chondromyxoid stroma of low cellularity (a: haematoxylin and eosin (H&E) stain). The tumour cell expresses immunohistochemically the endothelial marker CD31 (b) and shows nuclear expression of CAMTA1 protein (c), diagnostic of an epithelioid haemangioendothelioma (EHE).

To address pain symptoms, radiotherapy to the right paravertebral mass was planned; however, due to a lack of response to treatment, a CT-guided biopsy of the site was performed before proceeding. Histological analysis revealed epithelioid neoplastic cells (Fig. 1a), which expressed CD31, CD34, and ERG on immunohistochemistry, indicating endothelial differentiation (Fig. 1b). Nuclear expression of CAMTA1 protein confirmed an EHE (Fig. 1c).

**Figure 2:**
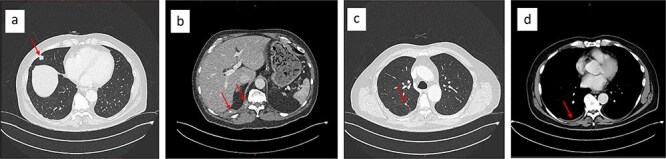
Chest CT showing partially calcified stable 15 × 13 mm subpleural lesion in the lateral middle lobe (a), a paravertebral mass at the level of the T11/12 vertebral foramen, and an intercostal lesion at the level of T10–12 (b). Nodular changes along the oblique fissure (c), and a mild right-sided pleural effusion (d).

Upon rituximab-bendamustin treatment completion, the CT-thorax/abdomen revealed complete NHL remission but demonstrated features consistent with thoracic EHE. The CT displayed multiple pulmonary nodules on the right, with partially calcified lesions, including subpleural (Fig. 2a), paravertebral, and intercostal lesions (Fig. 2b). Additionally, there were increasing nodular changes along the oblique fissure and a pleural effusion (Fig. 2d).

**Figure 3:**
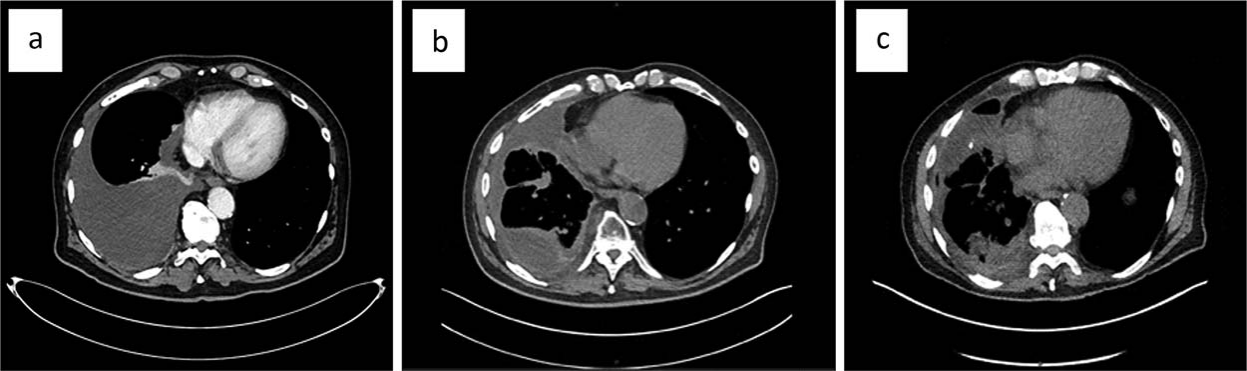
The evolution of the malignant pleural effusion over time. Progressively increasing pleural effusion ~4 months after first identified (a), a loculated pleural effusion after three repeat thoracocenteses, talc pleurodesis, and IPC placement (b); loculated pleural effusion about 2 months after VATS partial empyema evacuation (c).

Systemic therapy with pazopanib 800 mg (2-0-0-0) was initiated early on. The patient experienced recurrent pleural effusions, which cytology confirmed contained EHE cells. The malignant pleural effusion (MPE) demonstrated rapid reaccumulation and was initially managed with repeat thoracenteses. During the first thoracentesis, 2000 ml of pleural fluid was aspirated. Sixteen days later, 2705 ml was drawn, and 1800 ml just 8 days later. Eventually, a video-assisted thoracoscopic surgery (VATS) was performed at another hospital, which included talc pleurodesis, achieving full lung re-expansion, and prophylactic indwelling pleural catheter (IPC) placement. Unfortunately, the patient continued to develop recurrent MPEs (Fig. 3a), now loculated and accompanied by lung entrapment (Fig. 3b), markedly increasing the complexity of management.

The patient presented to our institution for further evaluation. We performed a VATS, achieving only partial evacuation of the empyema’s fibrinopurulent debris, due to excessive intraoperative bleeding (Fig. 3c). The histopathology of the visceral pleural fragments obtained showed no evidence of EHE. Two weeks later, we removed the chest drain with minimal effusion in the right costophrenic angle. The patient’s quality of life is still significantly reduced by his severe breathlessness, but after careful consideration, we decided against performing VATS decortication.

## Discussion

The management of EHE remains challenging due to its unpredictable behaviour, marked by years of stability in a considerable proportion of patients. Surgical resection remains the cornerstone of treatment in unifocal EHE [1]. However, given that most patients have multifocal primary disease at the time of diagnosis, and about 70% develop metastases, surgery is often not recommended [5].

MPEs are present in 20% of cases of pulmonary EHE [6]. Dyspnoea contributes significantly to their high symptom burden and reduced quality of life [7]. In patients with systemic symptoms or serosal effusions, systemic therapy and palliative care may be offered early on [1] but without delaying definitive pleural interventions [8].

Management strategies for MPE include thoracentesis or definitive pleural interventions, including IPCs, pleurodesis, or VATS decortication, which are preferred over repeat aspirations [8]. Definitive procedures are associated with fewer future pleural interventions and complications, including pneumothorax. Despite this, a study showed that only 24% of patients in whom the first recurrence occurred within 2 weeks received a definitive procedure. One contributing factor is that although physicians recognize recurrence as common, they may underestimate how quickly it may occur. Over half of MPEs recur within 2 weeks of initial thoracocentesis [9]. Therefore, timely referral for definitive pleural procedures is essential; it prevents repeat aspirations and worsening dyspnoea, optimizing both symptom control and improving quality of life.

The pleural effusions in pleural EHE are frequently loculated [10, 11], making drainage challenging. While adjuvant intrapleural fibrinolytic therapy (IFT) may be trialled to facilitate drainage in selected cases [8] of other malignancies, its use in EHE is not recommended. IFT is associated with a hypothetical increased risk of haemorrhage [12], which is possibly heightened given its vascular origin. Acknowledgement of this is important, as haemorrhagic MPEs can also reduce the effectiveness of talc pleurodesis [13].

IPC and pleurodesis are first-line interventions for expandable-lung MPEs. Talc pleurodesis may be combined with decortication surgery in non-expandable lung MPEs, as it may improve pleurodesis success [8]. VATS decortication has been reported to provide symptomatic relief of both dyspnoea and cough in all patients with trapped lung, and pleural fluid control in 96% of patients [14]. In a similar case report to ours, VATS decortication successfully managed the recurrent effusions, leading to symptomatic improvement and lower supplemental oxygen requirements [11].

## Conclusion

Treatment strategies, including pleurodesis and VATS decortication, should be considered for MPE management in EHE. Although VATS decortication was an appealing option in our patient, given the loculated pleural effusion, trapped lung, and the evidence supporting its role in relieving dyspnoea, it was ultimately not pursued. The patient’s limited surgical fitness and the surgical risks, most notably bleeding, are currently considered to outweigh any potential benefits.
